# Development and collaborative validation of an event-specific quantitative real-time PCR method for detection of genetically modified CC-2 maize

**DOI:** 10.3389/fpls.2024.1460038

**Published:** 2024-09-10

**Authors:** Likun Long, Ning Zhao, Congcong Li, Yuxuan He, Liming Dong, Wei Yan, Zhenjuan Xing, Wei Xia, Yue Ma, Yanbo Xie, Na Liu, Feiwu Li

**Affiliations:** Institute of Agricultural Quality Standard and Testing Technology, Jilin Academy of Agricultural Sciences, Changchun, China

**Keywords:** genetically modified maize CC-2, event-specific PCR, quantification, real-time quantitative PCR, detection, validation

## Abstract

As one of the developed genetically modified (GM) maize varieties in China, CC-2 has demonstrated promising commercial prospects during demonstration planting. The establishment of detection methods is a technical prerequisite for effective supervision and regulation of CC-2 maize. In this study, we have developed an event-specific quantification method that targets the junction region between the exogenous gene and the 5’ flanking genomic DNA (gDNA) of CC-2. The accuracy and precision of this method were evaluated across high, medium, and low levels of CC-2 maize content, revealing biases within ±25% and satisfactory precision data. Additionally, we determined the limits of quantification of the method to be 0.05% (equivalent to 20 copies) of the CC-2 maize. A collaborative trial further confirmed that our event-specific method for detecting CC-2 produces reliable, comparable, and reproducible results when applied to five different samples provided by various sources. Furthermore, we calculated the expanded uncertainty associated with determining the content level of CC-2 in these samples.

## 1 Introduction

Maize (*Zea mays L*.) occupies a significant position in the domain of genetically modified organisms (GMOs), with more than 25% of global maize varieties undergoing genetic alteration (ISAAA, 2021). As of 2023, it holds the record for the highest number of approved GM crop events, totalling 69.32 million hectares and the number of authorized maize events has reached 421 (GM AgbioInvestor Monitor, 2024). Maize accounts for 146 of these events (Meng et al., 2022). The cultivation and utilization of genetically modified maize for food or feed purposes is becoming increasingly widespread on a global scale (Turkec et al., 2015; Avsar et al., 2020).

Prior to the commercial release of a new GM event, it is widely acknowledged that regulatory must be conducted to assess their potential impacts on human, animal and environmental health (Akinbo et al., 2021; Giraldo et al., 2019). The incorporation of tracking and tracing tools for transgenic insertion is considered an indispensable component of the deregulation process (European Commission Regulation (EC) No.1830/2003). Furthermore, the development of detection methods for GM identification and quantification is not only pivotal for ensuring legality and traceability but also for compliance with GM labelling regulations (Gruère and Rao, 2007). Moreover, method validation plays a crucial role in standardizing GM testing methods to ensure that GM testing laboratories can generate reliable analytical results (Meng et al., 2022).

The GM maize CC-2, developed by the Chinese Agricultural University (CN Patent No. CN105331725A), is a transgenic maize event containing modified *EPSPS* genes linking with the the chloroplast signaling peptide of sorghum named *maroACC* gene (Chen et al., 2019; Fan et al., 2018). It is one of the first batch of GM maize varieties in China obtaining production safety certificates, and it will have great commercial potential. Therefore, developing highly specific event-specific PCR methods for GM maize CC-2 and its derivatives is of significant importance for promoting the commercialization of GM maize in China. At the same time, the application of this method will also provide necessary technical support for safety testing, intellectual property protection, and product supervision (Li et al., 2020). Therefore, there is an urgent need for more specific, accurate, and standardized methods to meet the technical requirements of GM regulation in various countries’ food trade regulations (Carzoli et al., 2018). Real-time PCR is widely regarded as the gold standard method for GM quantification in food or feed products (Li et al., 2022; Turkec et al., 2015; Avsar et al., 2020), though many other new GMO detection techniques such as microarray, digital PCR, re-sequencing, and biosensor, etc., were also reported (Yi et al., 2022; Li et al., 2017; Fraiture et al., 2021). The reliability of inter-laboratory results relies on method comparisons, validation, and harmonization. The inter-laboratory validation of methods is a crucial step in standardizing GM detection procedures, as it empowers the GM detection laboratory to generate dependable analytical outcomes.

This study established an event-specific real-time PCR method based on the molecular characteristics of CC-2 for the detection and quantification of GM maize CC-2. For this method, we organized a collaborative ring trial, which confirmed the specificity, applicability and viability of quantitative determination and the range of quantitative uncertainty. The development and application of the method will provide technical support for CC-2 maize commercialization supervision and implementation of quantitative labelling system.

## 2 Materials and methods

### 2.1 Plant material

Seeds of homozygous GM maize CC-2 and its non-GM maize ZD958 were provided by the Agricultural University of China. For specific testing, a total of 42 varieties of other GM crop events were graciously supplied by their respective developers, encompassing 13 transgenic maize (*Zea mays L.*) events (MON810, MON863, MON88017, MON89034, MON87460, MON87427, NK603, GA21, Bt176, Bt11, MIR604, MIR162, 3272, DAS-40278, 59122, 5307, 4114, T25, DBN9936, C0010.3.7 and TC1507), 7 transgenic soybean (*Glycine max L.*) events (GTS 40-3-2, A2704-12, MON89788, DP-356043, A5547-127, CV-127 and DP-305423), 7 transgenic rapeseed (*Brassica napus L.*) events (MS1, Topas19/2, Oxy-235, MS8, RF1/RF2/RF3 and T45) and 6 transgenic cotton (*Gossypium hirsutum L.*) events (MON531, MON88913, MON1445, MON15985, LLCotton25 and GHB614). All these seeds served as a source of genomic DNA for the purpose of this study.

### 2.2 Sample preparation

Seeds of CC-2 and non-GM maize were planted in the greenhouse. Genomic DNA extracted from leaves was used for quantitative DNA calibrant. Blind matrix samples containing different CC-2 event mass fractions (5%, 2%, 1%, 0.5%, and 0.1%) were created using ground seed powder from both types of maize, provided by the Development Center of Science and Technology, Ministry of Agriculture and Rural Affairs, China.

### 2.3 DNA extraction

The DNeasy Plant Mini Kit (Qiagen, Hilden, Germany) was used to extract genomic DNA (gDNA) from plant or seed material following the manufacturer’s instructions. The quality of the gDNA was evaluated by measuring the OD260/OD280 ratio with a Nanodrop 8000 (Thermo Scientific™ NanoDrop™, USA). To analyze low initial DNA concentrations, CC-2 maize DNA samples were diluted with water and prepared at concentrations of 10, 5, 0.4, 0.08, and 0.016 ng/µl using QubitR 2.0 (Life Technologies, United States). The copy number of gDNA was estimated based on the haploid genome size of maize being 2500 Megabasepairs (Arumuganathan and Earle, 1991), corresponding to a weight of 2.74 pg.

### 2.4 Primers and probes design

The design of primers and probes at the mutation positions was carried out using Primer Express Software 3.0 following the manufacturer’s instructions. The design principles involved placing one set of primers/probe on either the 5’ or 3’ side of the exogenous gene insertion locus in the genome, as well as specifying the region for the 5’ end of probes to maintain sensitivity. Candidate primer pairs were additionally confirmed through traditional endpoint PCR to ensure generation of a single PCR product of correct size. Endogenous gene probes were labelled with 5’HEX, while specific probes were labelled with 5’ FAM, both quenched with BHQ or MGB at the 3’end (Sangon BioTech, China). The details of the primers and probes used in this study are provided in **Table 1**.

**Table 1:** Primer/probe information of CC-2 and zSSIIb.

Purpose	Name	Sequence (5’-3’)	Amplicon size (bp)	Specificity	Source
PCR analysis of *zSSIIb* gene	zSSIIb-F	CGGTGGATGCTAAGGCTGATG	88	Maize genome	Yang et al., 2005
	zSSIIb-R	AAAGGGCCAGGTTCATTATCCTC			
	zSSIIb-P	HEX - TAAGGAGCACTCGCCGCCGCATCTG -BHQ1			
Event-specific PCR analysis of CC-2	CC-2-F	TGCAATGGGCCAGATCTAGTTA	107	5’ junction of CC-2 event	this study
	CC-2-R	GCTCACTGAATTAACGCCGA			
	CC-2-P	FAM - CCAGTACTAAAATCCAGATCCCCCGA -BHQ1			

### 2.5 Quantitative real-time PCR

The 7,500 Real-Time PCR System from Life Technologies AB (USA) was employed for quantitative real-time PCR analysis. The amplification system followed the instructions provided by Roche (Switzerland) for FastStart Universal Probe Master and ROX reference dye. qPCR analysis was conducted according to the methodology described in previous research (Guertler et al., 2019), with a minimum of three biological replicate samples included in each experiment. The maize endogenous gene *zSSIIb* (maize starch synthase IIb) and the event CC-2 specific fragment were separately amplified following the thermal cycle protocol: 95 °C for 5 min, 40 cycles at 95 °C for 15 s (denaturation), and 60 °C for 1 min (annealing and extension). Fluorescent signals were read out during the extension steps, and analyzed using the software Option Monitor 2 version 2.02 (MJ Research, Waltham, MA, USA).

### 2.6 Method verification

The validation of this method adheres to the standards outlined in the “ Verification of analytical methods for GMO testing when implementing interlaboratory validated methods” (Hougs et al., 2017). Key parameters including dynamic range, accuracy, precision, limit of detection (LOD) and limit of quantification (LOQ) were assessed. Furthermore, the specificity of the method was investigated by analyzing DNA samples from diverse species encompassing GM maize events as well as soybean, cotton, sugar beet, and rapeseed. Additionally, sensitivity in detecting target sequences in other DNA samples was evaluated by combining an equal amount of DNA from three GM maize samples with a positive control (CC-2 maize).

### 2.7 Collaborative trials

The ring trial comprised eight GMO detection laboratories, all of which were affiliated with the Ministry of Agriculture, China. Each laboratory was provided with seven genomic samples: one sample labelled as CC-2 was utilized for constructing standard curves through serial dilution; one sample designated as a negative control; and five blind samples labelled S1, S2, S3, S4 and S5 representing CC-2 content levels of 5%, 2%, 1%, 0.5% and 0.1% respectively. Each sample had a volume of 100 μL at a concentration of 50 ng/μL. The genomic DNA samples along with the primers/probe were stored in an enclosed container filled with dry ice and dispatched to each participating laboratory.

## 3 Results and discussion

### 3.1 Establishment of CC-2 event-specific PCR

**Figure 1:**
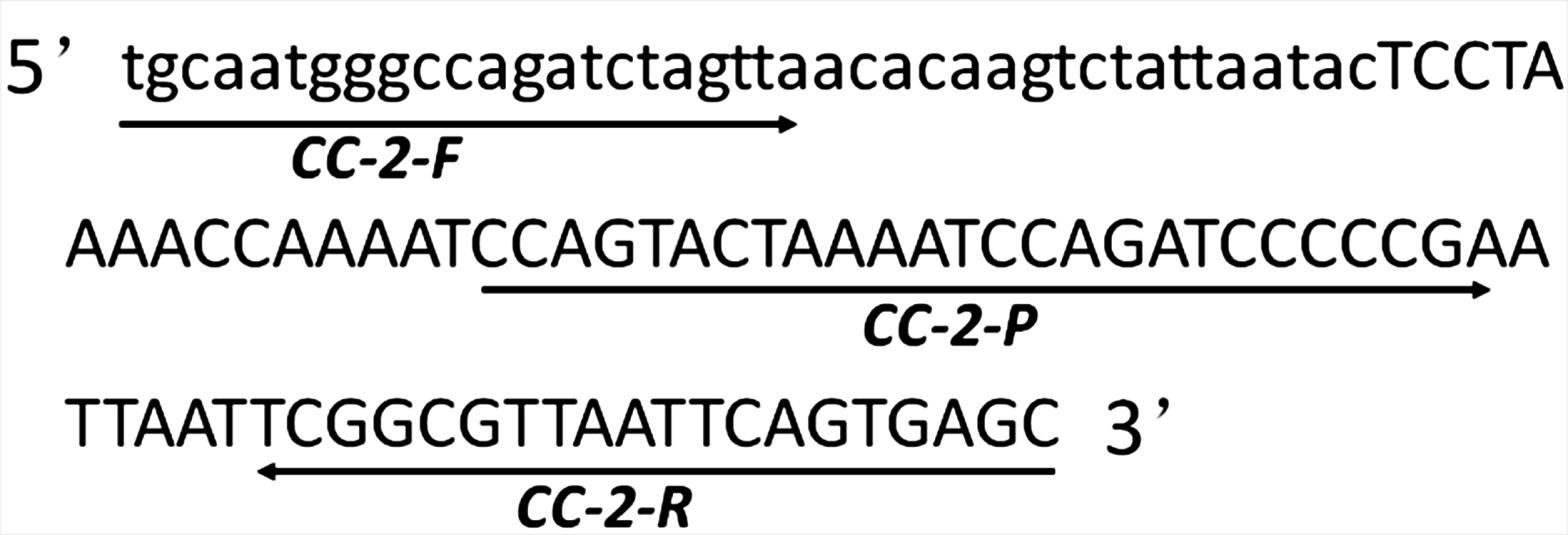
Amplicon sequence of GM maize CC-2 event-specific PCR. The junction fragment of CC-2 event covered 40 bp of flanking genomic DNA in lowercase letters and 67 bp exogenous insertion fragment in uppercase letters and the sequence of forward primer CC-2-F, reverse primer CC-2-R and probe CC-2-P were underlined.

The 5’ end of the insert and the flanking sequence of maize genomic DNA were provided and licensed by China Agricultural University for reference and method development (Patent No. CN201510856966). Blastn analysis against sequences in the GenBank database confirmed that the isolated junctions indeed spanned the integration border between the genome and integrated construct. Multiple primer-probe combinations specific to CC-2 were designed based on the 5’ end-boundary genome sequence and CC-2 insertion, utilizing online software Primer3 (http://primer3.ut.ee/). These primers and probes were screened through qPCR amplification using CC-2 genomic DNA as a template. Among them, CC-2-F/R combined with QP primer probes exhibited a characteristic “S” shaped curve with stronger amplification signal and lower quantification cycle (Cq) value, making it a potential candidate for further analysis. The amplified fragment length of this combination was determined to be 107 bp through sequencing verification, which matched expectations. As an endogenous reference gene in maize, *zSSIIb* gene was employed for quantitative PCR assay due to its confirmed single copy status in maize genome; moreover, real-time PCR protocol for *zSSIIb* gene had been previously validated (Yang et al., 2005). Specific information regarding primer probes is presented in **Figure 1** while details about primers and probes are provided in **Table 1**.

In order to optimize the real-time fluorescence PCR reaction system, concentration gradients of six primers (0 μmol/L, 0.1 μmol/L, 0.2 μmol/L, 0.4 μmol/L, 0.6 μmol/L and 0.8 μmol/L) were set respectively, with the probe concentration being half of the primer concentration. The concentrations of primers and probes were determined based on fluorescence curves and relative Cq values. The results demonstrated that the smallest Cq value and higher fluorescence intensity were achieved when using a primer concentration of 0.4 μmol/L and a probe concentration of 0.2 μmol/L; there was no significant difference compared to amplification with high-concentration primer-probe pairs (**Supplementary Figure S1**). Considering its consistency with the internal standard gene zSSIIb and minimal impact on annealing temperature in real-time fluorescent PCR reactions, this method adopted the conventional qPCR reaction procedure.

### 3.2 Specific test of CC-2 event-specific PCR method

The specific test of the CC-2 primer combination CC-2-uQF/uQR/uQP was conducted on different crops and their main commercial events. The samples consisted of 6 mixed samples of common GM crops and 6 mixed samples of non-GM maize. **Supplementary Table S1** provides detailed information on the events and results. The obtained results demonstrated that only the CC-2 sample exhibited the expected amplification curve, while other samples did not show such curves. These findings indicate that the method exhibits excellent specificity for detecting the CC-2 maize event.

### 3.3 LOD

The concentration of homozygous genomic DNA template was quantified and the copy numbers were calculated based on an estimated haploid maize genome size of 2500 Mbp (Arumuganathan and Earle, 1991). Six samples of the homozygous genomic DNA template were serially diluted to achieve copy number concentrations of 40, 20, 10, 5, 2.5 and 0.5 copies/μL. Subsequently, 2 μL test samples were introduced into the system. Utilizing a total DNA template amount of 100 ng, the mass-percentage of CC-2 event corresponded to approximately 0.20%, 0.10%, 0.05%, 0.25%, 0.125% and 0.025%. Each dilution was subjected to analysis in 12 parallel reactions to identified LOD.

**Figure 2:**
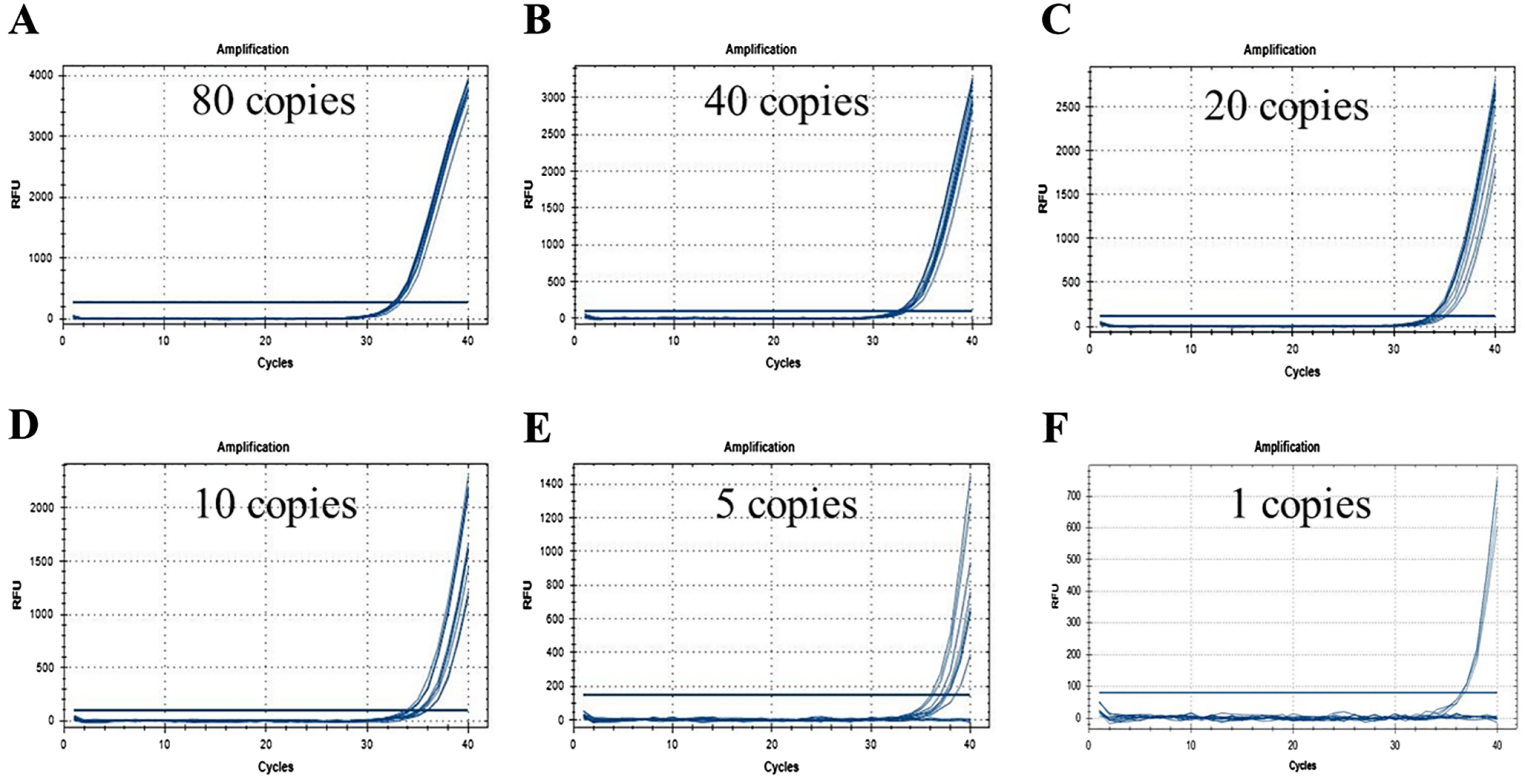
LOD test of CC-2 event specific real-time PCR method. is the amplification of 10 replicates when the amount of substrate in the reaction system is 80 **(A)**, 40 **(B)**, 20 **(C)**, 10 **(D)**, 5 **(E)** and 1 **(F)** copy, respectively.

The amplification test results are presented in **Figure 2**, and the statistical findings are summarized in **Table 2**. The data indicate that at a template concentration of 0.5 copies/μL, corresponding to 1 copy of substrate in the reaction system, 5 out of 12 parallel reactions yielded positive results, validating the accuracy of the template concentration dilution. At a template volume of 5 copies, 9 out of 12 parallel reactions were positive. When using templates with 2 copies, 7 out of 12 parallel reactions produced a positive result, but mean copy number was not available. Notably, typical amplification curves were obtained for all twelve parallel reactions when utilizing templates with copy numbers ranging from 10 to 40 copies. These observations suggest that the sample containing a fraction as low as 0.05% of CC-2 event (equivalent to 20 copies of CC-2 maize) could be reliably detected.

**Table 2:** LOD and LOQ of CC-2 event-specific realtime PCR method.

Amount of DNA (copies/reaction)	Signal rate (positive signals)	Mean Copy Number	SD of the Copy Number	RSD(%)	Bias(%)
40	12/12	36.31	7.3	23.10	-9.22
20	12/12	23.70	9.6	31.14	18.50
10	12/12	6.81	4.2	42.77	31.90
5	9/12	3.8	^b^	^b^	^b^
2	7/12	^b^	^b^	^b^	^b^
1	5/12	^b^	^b^	^b^	^b^

b Not available.

### 3.4 Dynamic range

**Figure 3:**
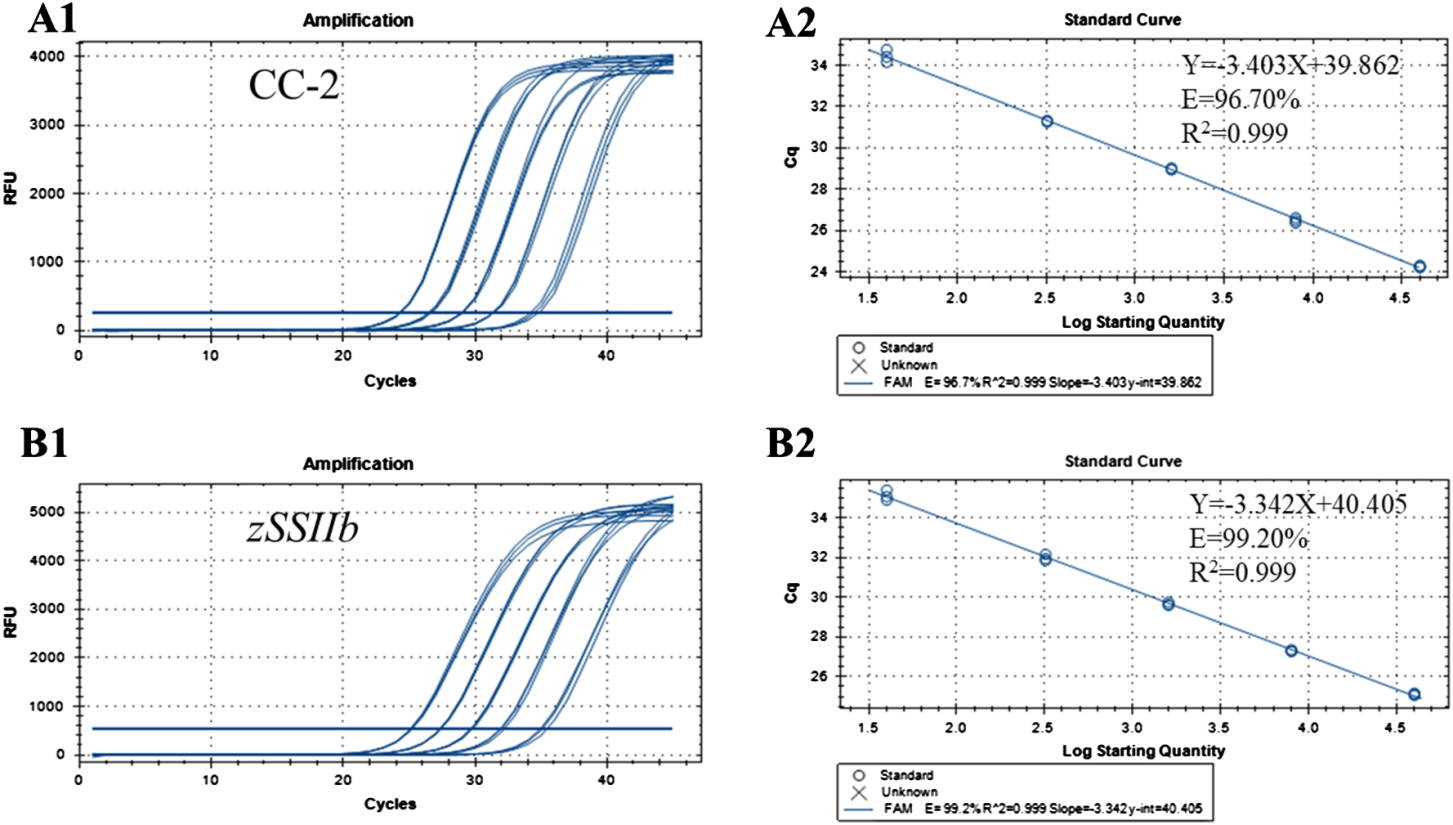
Amplification and standard curves for the event-specific quantitative PCR method using gradient-diluted CC-2 genomic DNA as the template analyzed using CFX96 System. **(A1)** Amplification graph for the CC-2 event-specific assay. **(A2)** Standard curve for the CC-2 event-specific assay. The copy numbers of the CC-2 event in each dilution were 40000,10000,1000,200 and 20 copies per reaction, respectively. **(B1)** Amplification graph for the endogenous gene *zSSIIb* assay. **(B2)** Standard curve for the gene *zSSIIb* assay. The quantities of maize genome in each dilution were 40000, 10000, 1000, 200, and 20 copies per reaction, respectively.

The concentration of genomic DNA was diluted to 50 ng/μl, followed by gradient dilution with water or 0.1×TE buffer to prepare multiple calibrators with varying contents. These calibrators were utilized as templates for real-time fluorescent PCR reactions, with the amount of DNA template in the PCR system being 2 μL. The corresponding DNA quality and copy number were then determined and are presented in **Table 3**. Standard curves for CC-2 and *zSSIIb* genes in maize were plotted based on the Cq value of PCR reaction of standard DNA solution and the logarithm of initial template copy number. The results indicated that when the calibrator ranged from 40 to 40000 copies, the slopes of the standard curves for CC-2 and *zSSIIb* in maize were -3.403 and -3.342 respectively (**Figure 3**). The coefficient of determination R^2^ was 0.999 for both cases, exceeding the minimum acceptable value of 0.98. The PCR amplification efficiencies were 96.70% and 99.20%, respectively, falling within the permissible range of 90% to 110%. More than three replicates were conducted, and all data parameters of standard curves met the requirements for quantitative GM detection methods. The PCR reaction system exhibited a good linear relationship between the PCR Cq value and the copy number detection of CC-2 specific fragment.

**Table 3:** Repeatability of real-time PCR assays employing CC-2 DNA as reference.

DNA amount (ng)	CC-2 copy number^a^	Repeat	Cq			Mean of Cq Values	SD_r_	RSD_r_ (%)^c^	Mean of all Cq Values	SD_R_	RSD_R_ (%)^c^
			1	2	3						
100	40000	1	24.24	24.31	24.22	24.26	0.05	0.19	24.39	0.11	0.45
		2	24.41	24.47	24.39	24.42	0.04	0.17			
		3	24.5	24.53	24.4	24.48	0.07	0.28			
20	8000	1	26.38	26.49	26.62	26.50	0.12	0.45	26.65	0.15	0.56
		2	26.67	26.58	26.68	26.64	0.06	0.21			
		3	26.8	26.86	26.73	26.80	0.07	0.24			
4	1600	1	29.02	28.97	28.96	28.98	0.03	0.11	29.16	0.17	0.59
		2	29.16	29.16	29.18	29.17	0.01	0.04			
		3	29.27	29.51	29.24	29.34	0.15	0.50			
0.8	320	1	31.33	31.27	31.28	31.29	0.03	0.10	31.47	0.16	0.50
		2	31.57	31.47	31.55	31.53	0.05	0.17			
		3	31.53	31.49	31.75	31.59	0.14	0.44			
0.1	40	1	34.41	34.76	34.16	34.44	0.30	0.88	34.65	0.49	1.41
		2	35.17	34.29	34.79	34.75	0.44	1.27			
		3	34.54	34.14	35.61	34.76	0.76	2.19			
0.0125	5	1	36.25	^b^	36.01	36.96	^b^	^b^	36.91	0.97	2.62
		2	^b^	37.32	35.67	38.45	^b^	^b^			
		3	37.30	38.27	35.89	37.15	0.72	1.20			
0.0025	1	1	^b^	37.59	37.29	38.4	^b^	^b^	37.73	0.67	1.77
		2	^b^	^b^	38.71	38.71	^b^	^b^			
		3	37.33	^b^	^b^	37.33	^b^	^b^			

a Calculated based on a haploid maize genome size of 2500 Mbp.

b Not available.

c RSDr, Repeatability relative standard deviation; RSD_R_, Reproducibility relative standard deviation.

### 3.5 LOQ

The initial determination of the limit of quantification (LOQ) is established based on the concentration range of the low content calibrator as measured by the limit of detection (LOD) (see **Table 2**). The findings indicate that only when the substrate concentration in the sample reaches 40 copies, both the relative bias and relative standard deviation are ≤25%, falling within an acceptable range. Therefore, it can be concluded that a minimum substrate amount of 40 copies is required for accurate determination.

For the samples with the copy number fraction of 0.1% of CC-2 event that passed the preliminary test, a total of 60 quantitative tests were conducted to calculate the relative bias (biasR) and relative standard deviation (RSD) of the detection data (**Supplementary Figure S2**). The results showed that both the relative bias and relative standard deviation for all test samples fell within ±25% of the acceptable range, indicating that the quantitative limit of CC-2 fluorescent quantitative PCR method could be determined to reach 0.1%, thus meeting EU standards requirements (European Network of GMO Laboratories (ENGL), 2015).

### 3.6 Quantification of blind samples by real-time quantitative PCR

Samples containing genomic DNA with copy number fractions of 5%, 1%, and 0.1% of CC-2 were subjected to testing, with three parallel samples set for each level and the experiment repeated thrice. The proportion of CC-2 DNA to total maize DNA (%) was computed as (mean copies of GM maize of three parallel assays)/(mean copies of total maize DNA of three parallel assays)×100% (Kuribara et al., 2002). **Table 4** presents results indicating values of 5.23%, 1.00%, and 0.10% for the three respective samples. The relative bias (biasR) between the measured total average value and the nominal value of the three subsamples ranges from 0.37% to 4.51%, falling within the acceptable range of ±25%, indicating that the measurement results obtained using the CC-2 quantitative PCR method exhibit good accuracy. The relative standard deviation (RSD_r_) for repeatability of the three horizontal samples ranged from 1.06% to 14.38%, all of which were below the specified threshold of 25%. The results of repeatability analysis demonstrate that the CC-2 converter fluorescence quantitative PCR method exhibits excellent precision.

**Table 4:** Trueness and precision data for the CC-2 event-specific realtime PCR method.

Theoretical contents	Assay	Experimental (copies)			Mean(copies)	RSD_r_ (%)	Experimental (%)	Bias (%)	RSD_R_ (%)
		1	2	3					
5.00%	CC-2	2001	2093	1944	2013	3.74	5.23	4.51	4.97
	*zSSIIb*	36597	39867	39237	38567	4.50			
1.00%	CC-2	371	389	411	390	5.13	1.00	0.37	1.06
	*zSSIIb*	36483	39197	41050	38910	5.90			
0.10%	CC-2	41	41	31	38	15.33	0.10	2.44	14.38
	*zSSIIb*	35763	37867	35693	36441	3.39			

RSD_r_, Repeatability relative standard deviation; RSD_r_, Reproducibility relative standard deviation.

### 3.7 Collaborative validation of the quantitative PCR method for GM event CC-2 detection

The new qualitative detection concept would be useful for ensuring robust and reproducible results among laboratories, particularly for detecting low-copy-number DNA samples. Samples of different CC-2 content were performed an interlaboratory evaluation of the developed quantitative method as a blind test performed by 8 laboratories. Blind samples with the CC-2 content levels of 5%, 2%, 1%, 0.5% and 0.1% were prepared and provided to measure in each lab. Samples of one content has 3 sub-samples.

As the result submitted of 8 labs shown (**Supplementary Table S2**; **Figure S3**), the slope of standard curves of CC-2 specific and *zSSIIb* gene in eight laboratories ranged from -42.63 to -38.29 and -42.95 to -38.091, respectively. The determination coefficients R^2^ ranged from 0.995 to 1.000 and from 0.992 to 1.000, respectively, PCR amplification efficiency ranged from 91.70 to 106.14% and from 90.09 to 101.4%, respectively. All the tests of the standard curves were within the acceptable range.

For the different content samples measured data, Cochran’s test (p<0.025) and Grubb’s test (p<0.025) were carried according to the harmonized guidelines of AOAC to remove the outlier data and analyze the validated result. The results show that there are no outliers or deviations in 8 laboratory data. the data of 15 samples with 3 different content from 8 laboratories were statistically summarized, and the average value from 8 laboratories was calculated (**Table 5**). The average values of 5 content samples in laboratories were 4.81%, 2.18%, 0.96%, 0.51% and 0.095%, respectively. The quantitative values deviated slightly from the expected values for all tested samples with bias (%) ranging from -5.0% and 9.0%. In the ENGL method acceptance criterion, the trueness should be within ±25% (European Network of GMO Laboratories (ENGL), 2015). It indicated that the CC-2 PCR specific method in quantitative measurement was credible.

**Table 5:** Determined GM% values of the eight participants for the five unknown samples.

Labs		GMO content (GM% = GM copy number/genome copy number × 100)(%)				
		Level 1	Level 2	Level 3	Level 4	Level 5
Lab 1	Rep 1	3.90	2.26	0.66	0.49	0.09
	Rep 2	4.05	2.44	0.71	0.52	0.08
	Rep 3	4.06	2.27	0.71	0.51	0.08
Lab 2	Rep 1	4.67	2.49	0.84	0.38	0.08
	Rep 2	4.45	2.31	0.81	0.6	0.07
	Rep 3	4.29	2.45	0.85	0.51	0.08
Lab 3	Rep 1	5.15	2.06	0.97	0.49	0.10
	Rep 2	5.24	2.10	1.00	0.5	0.10
	Rep 3	5.07	1.99	0.95	0.5	0.09
Lab 4	Rep 1	4.92	2.02	0.88	0.53	0.10
	Rep 2	4.99	2.07	0.87	0.52	0.11
	Rep 3	4.93	2.00	0.93	0.5	0.10
Lab 5	Rep 1	5.66	1.83	1.17	0.47	0.11
	Rep 2	5.90	1.99	1.08	0.50	0.10
	Rep 3	5.53	1.93	1.13	0.48	0.10
Lab 6	Rep 1	5.40	2.37	1.09	0.55	0.12
	Rep 2	4.29	2.30	1.13	0.51	0.10
	Rep 3	4.43	2.47	1.14	0.48	0.08
Lab 7	Rep 1	4.96	2.03	1.02	0.53	0.10
	Rep 2	4.59	2.01	0.97	0.53	0.10
	Rep 3	5.00	2.02	1.07	0.54	0.10
Lab 8	Rep 1	4.53	2.41	0.961	0.53	0.11
	Rep 2	4.51	2.35	1.036	0.47	0.11
	Rep 3	4.97	2.28	1.123	0.55	0.10
**Mean value**		**4.81**	**2.18**	**0.96**	**0.51**	**0.095**

After that, we conducted further statistical analyses for the values of quantification. The trueness and precision were determined as previously described. The mean, bias, repeatability of RSD (RSD_r_) and reproducibility of RSD (RSD_R_) of blind samples were measured (**Table 6**). The RSD_r_ values for samples were 11.43%, 8.44%, 15.73%, 3.38% and 8.75%, respectively; all RSD^r^ values were below 25%. The RSD_R_ values were within the range of 3.23% to 12.19%, all below 35% across the entire dynamic range. Both the RSD of test samples were similar to or within a narrower range than those in previously reported method of GMO events (Wang et al., 2013; Mano et al., 2013; Jacchia et al., 2015). The repeatability and reproducibility of the method meet the acceptance criteria and performance requirements (European Network of GMO Laboratories (ENGL), 2015), indicating that the CC-2 specific method is stable, reliable, and suitable for quantifying CC-2. The analysis results demonstrate that the established event-specific real-time PCR system for CC-2 can generate accurate, repeatable, and comparable results across different laboratories.

**Table 6:** Summary of validation results for the CC-2-specific method.

blind Samples	Expected value(%)				
	5.0	2.0	1.0	0.5	0.1
Mean value	4.81	2.18	0.96	0.51	0.095
repeatability standard deviation *Sr*	0.558	0.179	0.151	0.017	0.009
relative repeatability standard deviation, *RSD_r_* (%)	11.43	8.44	15.73	3.38	8.75
reproducibility standard deviation, *S_R_*	0.59	0.18	0.15	0.017	0.009
relative reproducibility standard deviation, *RSD*_R_ (%)	12.19	8.50	15.81	3.23	8.77
bias (absolute value)	-0.19	0.18	-0.04	0.01	-0.005
*biasR* (%)	-3.80	9.00	-4.00	2.00	-5.00

### 3.8 Measurement uncertainty of the tested results

**Figure 4:**
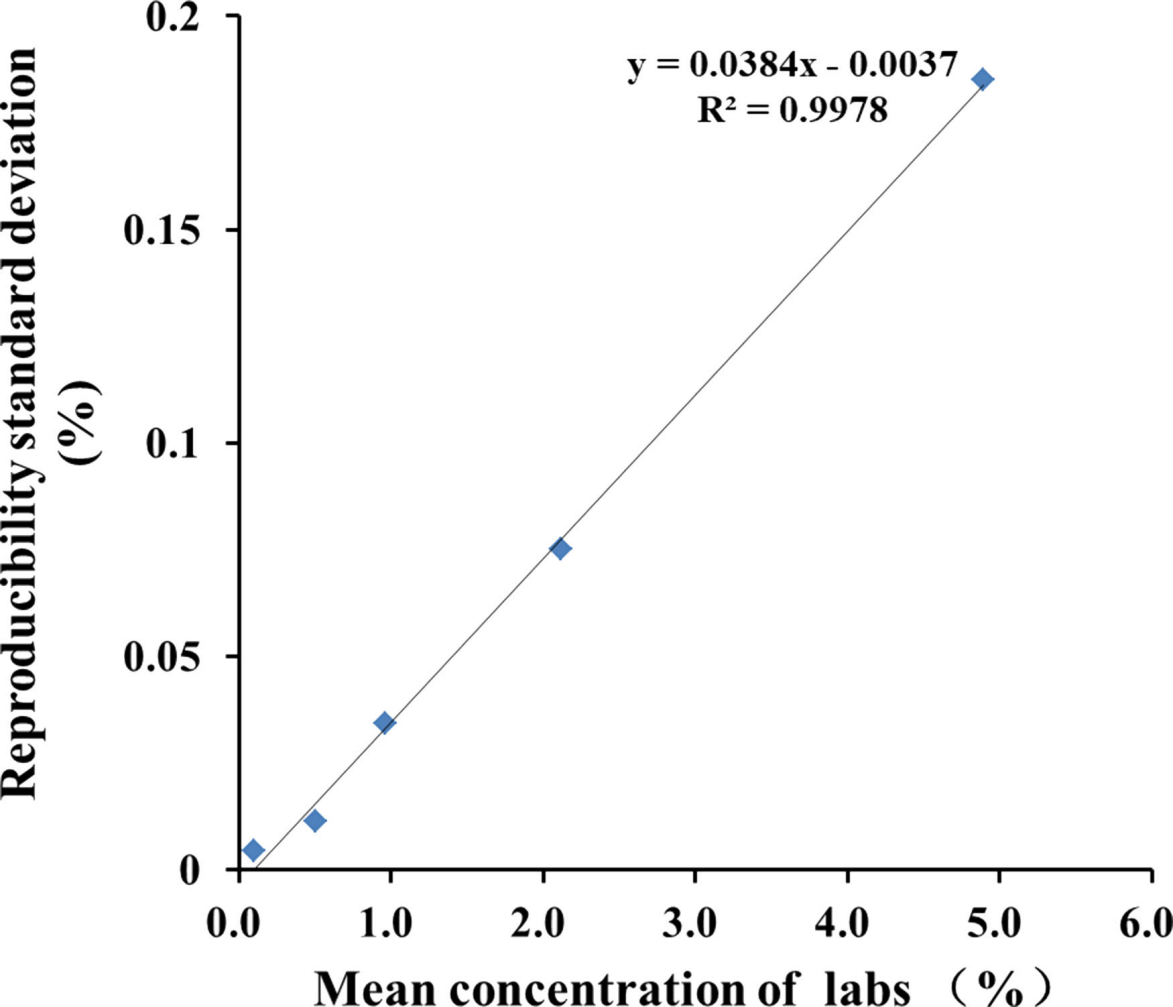
Linear regression produced by plotting mean measurement concentration (c) against reproducibility standard deviation (sR).

According to the guidance documents (Trapmann et al., 2007; Standardization ISO/TS 21748:2004), an estimation of measurement uncertainty (MU) was conducted for the quantitative results. This can be achieved by plotting a chart correlating repeatability standard deviation (sR value) in collaborative trials with the average number of blind samples tested (c), and calculating linear regression to estimate absolute standard uncertainty (u0) and relative standard uncertainty (RSU). The value of u0 is a constant equal to the intercept of linear regression (u0 = 0.0037), while RSU is equal to the slope of linear regression (RSU = 0.0384). The critical value (LC = 2 × u0) corresponds to a measurement result of 0.1% CC-2, indicating that if the estimated value is below 0.1%, it can be concluded with a confidence level of 95% that target CC-2 does not exist in the tested sample. The standard uncertainty associated with measurement results (u) is calculated using formula u = (0.0037^2^ + (0.0384 × c)^2^) ^1/2^) (**Figure 4**). Typically, when reporting test results, an accompanying measurement uncertainty is provided as expanded uncertainty (U= 2 × u), which is derived from standard uncertainty using a coverage factor of 2.This corresponds approximately to a confidence level of about 95%. For blind samples S1-S5, respective values for c in measurement results are 5.083%, 2.071%, 1.049%, 0.503% and 0.102% (w/w).The U values for expanded uncertainties were calculated as follows: S1-0.144%, S2- 0.059%, S3-0.030%, S4-0.013% and S5-0.007%(w/w). Here, the uncertainty formula provides a closely approximate representation of the true distribution when laboratories utilize the CC-2 quantitative PCR method, offering a suitable level of precision for scientific research and analysis.

## 4 Conclusion

In this study, a novel real-time PCR-based analytical method was developed for the event-specific quantification of a genetically modified (GM) maize event CC-2. The specificity, sensitivity of these methods were determined with different concentrations of GM mixing samples. The LODs of these methods for CC-2 segment calculated as the amount of CC-2 were 0.05% or less. The limit of quantitation for the method was estimated to be 0.1% indicating that the LOQ of CC-2 was lower than 40 copies of maize haploid genome. The quantitative method was evaluated by means of blind tests in multi-laboratory trials. The trueness and precision were evaluated as the bias and reproducibility of relative standard deviation (RSD), and the determined bias and RSD values for the method were each less than 25%. These results suggest that the developed method would be suitable for practical analyses for the detection and quantification of CC-2. Furthermore, The uncertainty evaluation equation of the CC-2 method was established by the results from inter-laboratory verification to model the uncertainty arising from the relative repeatability standard deviation of inter-laboratory test values.

## Data Availability

The original contributions presented in the study are included in the article/**Supplementary Material**. Further inquiries can be directed to the corresponding author.
