# Supplementary material for: Development and collaborative validation of an event-specific quantitative real-time PCR method for detection of genetically modified CC-2 maize
Source: Front Plant Sci. 2024 Sep 10;15:1460038. doi: 10.3389/fpls.2024.1460038 (PMC11420048; doi:10.3389/fpls.2024.1460038)
Supplement: Supplementary file 1 [file DataSheet1.pdf]

Supplementary Figures

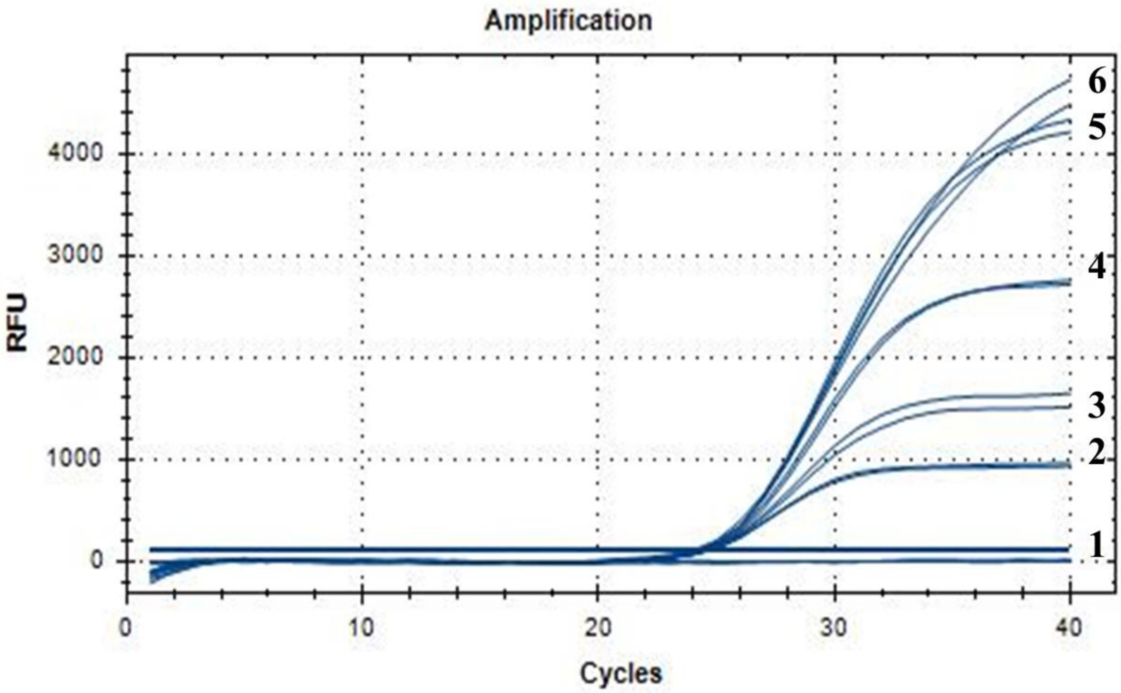

S1. Optimization of primer and probe concentration in real-time fluorescence amplification system. 1-6 were the amplification of CC-2 specific fragments when the final primer concentration was 0, 0.1, 0.2, 0.4, 0.6, 0.8 μM and the probe was half primer concentration, respectively.

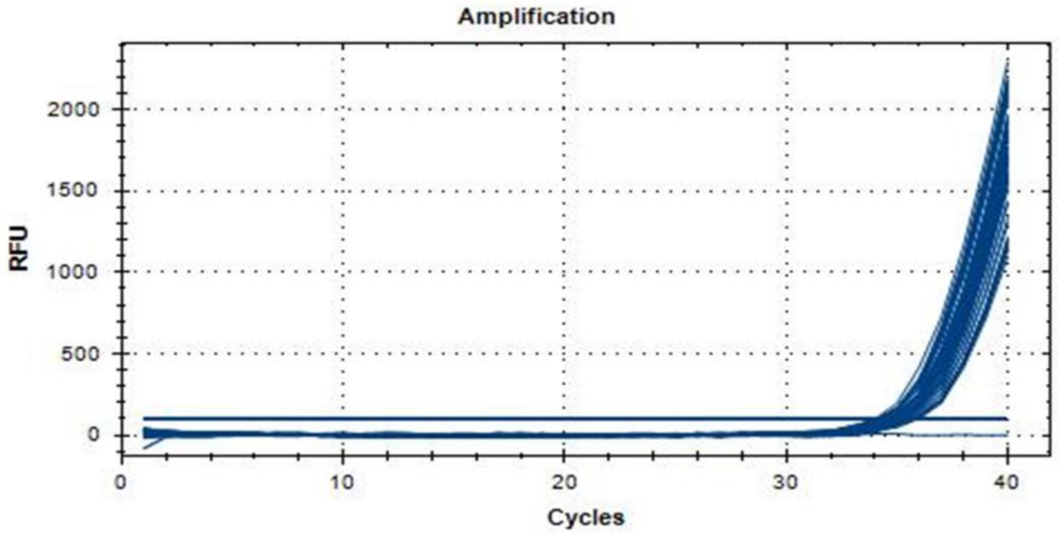

S2. Repeated determination by real-time PCR method on 60 samples of detection limit.

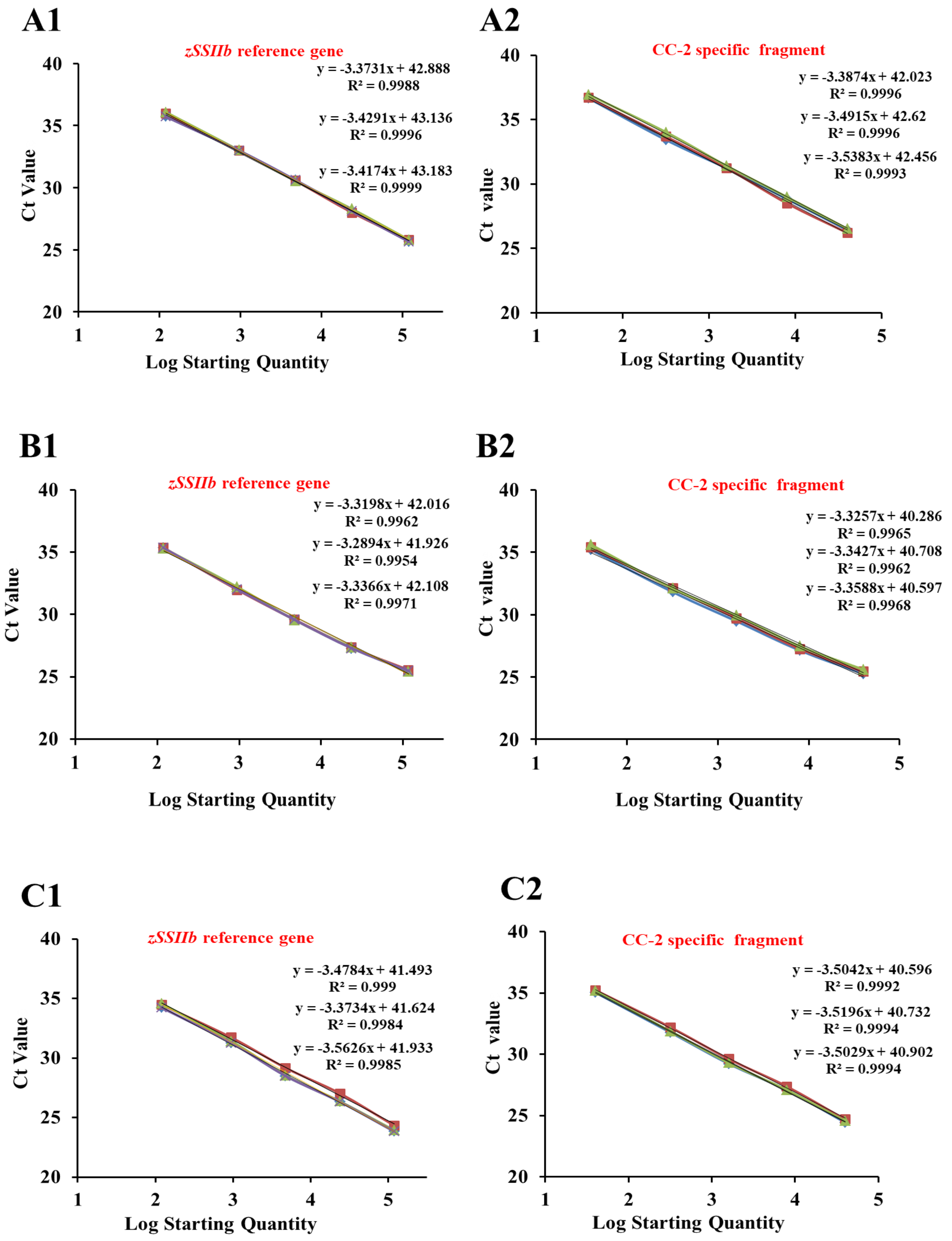

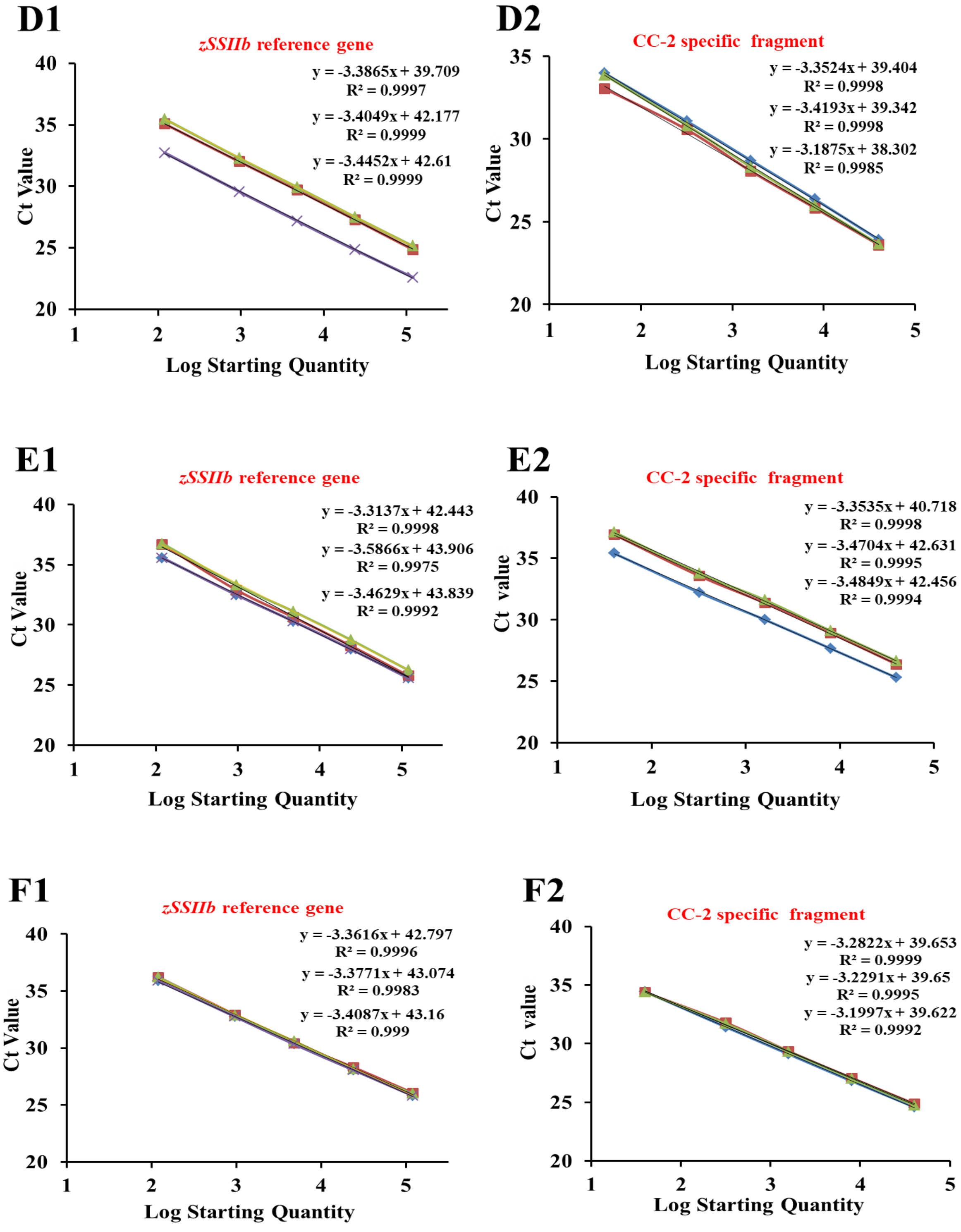
D1
zSSIIb reference gene
y = -3.3865x + 39.709
R² = 0.9997
y = -3.4049x + 42.177
R² = 0.9999
y = -3.4452x + 42.61
R² = 0.9999
Ct Value
Log Starting Quantity
D2
CC-2 specific fragment
y = -3.3524x + 39.404
R² = 0.9998
y = -3.4193x + 39.342
R² = 0.9998
y = -3.1875x + 38.302
R² = 0.9985
Ct Value
Log Starting Quantity
E1
zSSIIb reference gene
y = -3.3137x + 42.443
R² = 0.9998
y = -3.5866x + 43.906
R² = 0.9975
y = -3.4629x + 43.839
R² = 0.9992
Ct Value
Log Starting Quantity
E2
CC-2 specific fragment
y = -3.3535x + 40.718
R² = 0.9998
y = -3.4704x + 42.631
R² = 0.9995
y = -3.4849x + 42.456
R² = 0.9994
Ct value
Log Starting Quantity
F1
zSSIIb reference gene
y = -3.3616x + 42.797
R² = 0.9996
y = -3.3771x + 43.074
R² = 0.9983
y = -3.4087x + 43.16
R² = 0.999
Ct Value
Log Starting Quantity
F2
CC-2 specific fragment
y = -3.2822x + 39.653
R² = 0.9999
y = -3.2291x + 39.65
R² = 0.9995
y = -3.1997x + 39.622
R² = 0.9992
Ct value
Log Starting Quantity

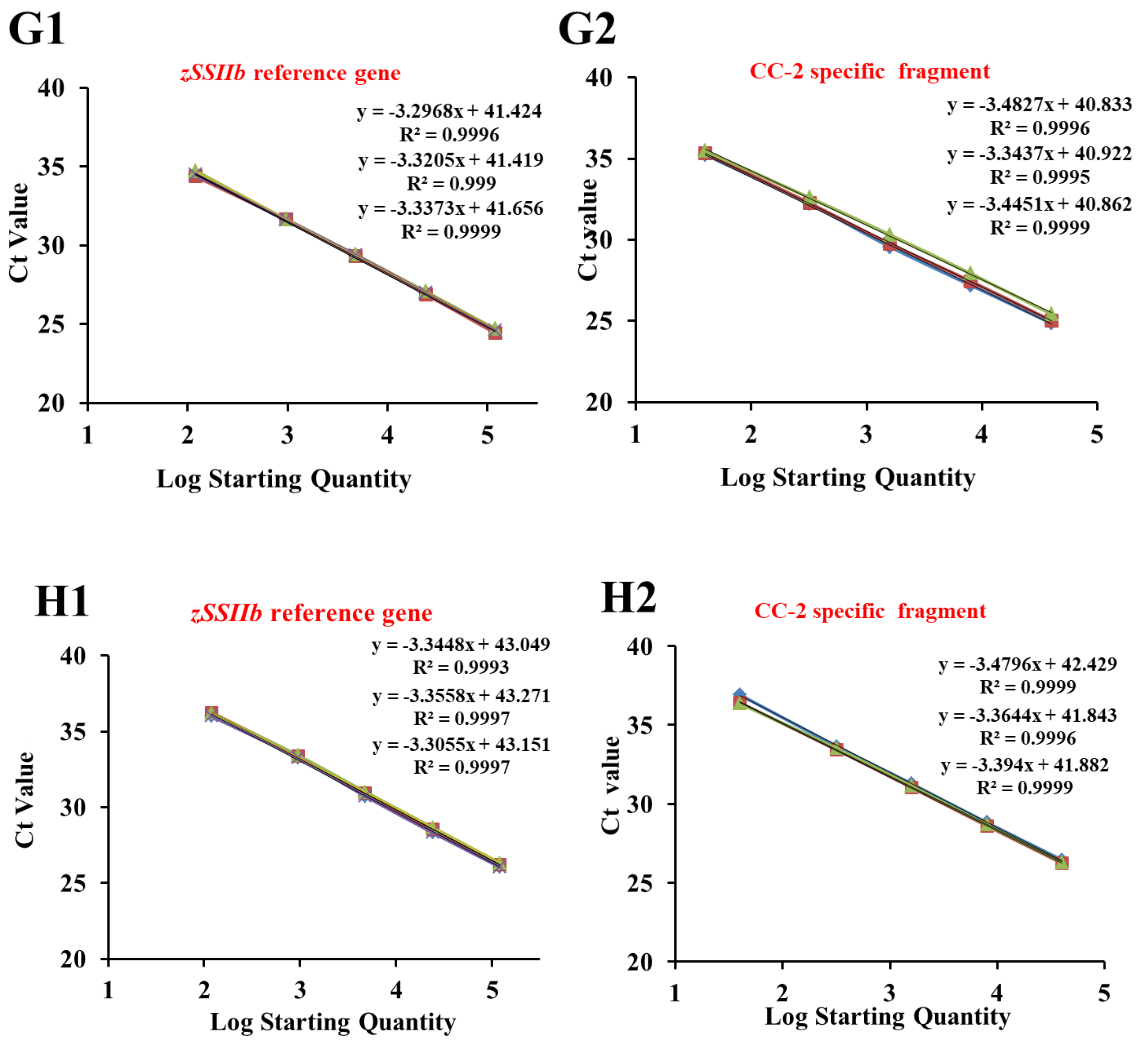

S3. Separate standard curves for the maize *zSSIIb* reference sequence and the CC-2 target sequence and were established by plotting Ct values against the log values of initial template copies in the PCR reactions, using serially diluted reference solutions from 100% CC-2 material. LabA1−LabH2 correspond to *zSSIIb* and CC-2 gene standard curves constructed according to the calibration Ct values provided by eight participating laboratories; each laboratory carried out three replications for the *zSSIIb* and CC-2 assays.
