# Supplementary material for: Development and collaborative validation of an event-specific quantitative real-time PCR method for detection of genetically modified CC-2 maize
Source: Front Plant Sci. 2024 Sep 10;15:1460038. doi: 10.3389/fpls.2024.1460038 (PMC11420048; doi:10.3389/fpls.2024.1460038)
Supplement: Supplementary file 2 [file Table1.docx]

**Table S1. Specificity test of the new qPCR assay**

| Species | Line/variety | Amplification | Expected result |
| --- | --- | --- | --- |
| Maize | CC-2 | + | + |
|  | Bt11 | - | - |
|  | Bt176 | - | - |
|  | MON810 | - | - |
|  | MON863 | - | - |
|  | NK603 | - | - |
|  | T25 | - | - |
|  | TC1507 | - | - |
|  | 59122 | - | - |
|  | MON89034 | - | - |
|  | MIR604 | - | - |
|  | MIR162 | - | - |
|  | 3272 | - | - |
|  | DAS-40278 | - | - |
|  | GA21 | - | - |
|  | MON88017 | - | - |
|  | MON87460 | - | - |
|  | 5307 | - | - |
|  | MON87427 | - | - |
|  | 4114 | - | - |
|  | DBN9936 | - | - |
|  | C0010.3.7 | - | - |
|  | Non-GM maize ZD958 | - | - |
| Rapeseed | MS1 | - | - |
|  | MS8 | - | - |
|  | RF1/RF2/RF3 | - | - |
|  | Oxy235 | - | - |
|  | T45 | - | - |
|  | Topas 19/2 | - | - |
| Soybean | GTS 40-3-2 | - | - |
|  | MON89788 | - | - |
|  | A2704-12 | - | - |
|  | A5547-127 | - | - |
|  | DP-305423 | - | - |
|  | DP-356043 | - | - |
|  | CV127 | - | - |
| Cotton | MON1445 | - | - |
|  | MON531 | - | - |
|  | MON15985 | - | - |
|  | LLCotton25 | - | - |
|  | MON88913 | - | - |
|  | GHB614 | - | - |

+, positive result; -, negative result.

**Table S2. Slope, PCR Efficiency, and R2 Values of the Standard Curves**

| **Labs** | **PCR** | **CC-2** | | | | ***zSSIIb* gene** | | |
| --- | --- | --- | --- | --- | --- | --- | --- | --- |
|  |  | **slope** | **linearity (R^2^)** | **PCR efficiency（%）** | **slope** | | **linearity (R^2^)** | **PCR efficiency （%）** |
| **Lab 1** | 1 | -3.42 | 0.999 | 96.10 | -3.44 | | 1.00 | 95.21 |
|  | 2 | -3.18 | 0.998 | 106.14 | -3.41 | | 1.00 | 96.62 |
|  | 3 | -3.36 | 0.998 | 98.42 | -3.39 | | 0.999 | 97.39 |
| **Lab 2** | 1 | -3.49 | 0.999 | 93.38 | -3.42 | | 0.999 | 96.16 |
|  | 2 | -3.54 | 0.997 | 91.70 | -3.43 | | 0.998 | 95.72 |
|  | 3 | -3.39 | 0.999 | 97.34 | -3.37 | | 0.997 | 97.92 |
| **Lab 3** | 1 | -3.34 | 0.995 | 99.10 | -3.34 | | 0.995 | 99.43 |
|  | 2 | -3.36 | 0.996 | 98.41 | -3.29 | | 0.995 | 101.41 |
|  | 3 | -3.33 | 0.995 | 99.82 | -3.32 | | 0.995 | 100.12 |
| **Lab 4** | 1 | -3.36 | 0.999 | 98.33 | -3.31 | | 0.999 | 100.70 |
|  | 2 | -3.39 | 0.999 | 97.05 | -3.36 | | 0.999 | 98.65 |
|  | 3 | -3.48 | 0.999 | 93.80 | -3.35 | | 0.997 | 99.35 |
| **Lab 5** | 1 | -3.23 | 0.999 | 104.04 | -3.41 | | 0.997 | 96.53 |
|  | 2 | -3.20 | 0.996 | 105.37 | -3.38 | | 0.997 | 97.77 |
|  | 3 | -3.29 | 0.999 | 101.29 | -3.36 | | 0.998 | 98.38 |
| **Lab 6** | 1 | -3.47 | 0.996 | 94.15 | -3.46 | | 0.998 | 94.41 |
|  | 2 | -3.49 | 0.998 | 93.63 | -3.59 | | 0.995 | 90.09 |
|  | 3 | -3.35 | 0.999 | 98.66 | -3.31 | | 0.999 | 100.38 |
| **Lab 7** | 1 | -3.34 | 0.999 | 99.10 | -3.34 | | 0.999 | 99.44 |
|  | 2 | -3.45 | 1.00 | 95.11 | -3.34 | | 0.999 | 99.38 |
|  | 3 | -3.47 | 0.999 | 94.51 | -3.35 | | 0.999 | 98.79 |
| **Lab 8** | 1 | -3.52 | 0.996 | 92.33 | -3.56 | | 0.992 | 91.96 |
|  | 2 | -3.50 | 0.995 | 93.51 | -3.37 | | 0.997 | 97.95 |
|  | 3 | -3.50 | 0.999 | 92.99 | -3.48 | | 0.994 | 93.87 |
